# Redefining the Distributional Boundaries and Phylogenetic Relationships for Ctenomids From Central Argentina

**DOI:** 10.3389/fgene.2021.698134

**Published:** 2021-08-04

**Authors:** Cecilia Soledad Carnovale, Gabriela Paula Fernández, Mariano Lisandro Merino, Matías Sebastián Mora

**Affiliations:** ^1^Centro de Bioinvestigaciones (CeBio), Universidad Nacional del Noroeste de la Provincia de Buenos Aires (UNNOBA-CICBA) / Centro de Investigaciones y Transferencia del Noroeste de la Provincia de Buenos Aires CITNOBA (UNNOBA-CONICET), Pergamino, Argentina; ^2^Comisión de Investigaciones Científicas de la Provincia de Buenos Aires (CICBA), La Plata, Argentina; ^3^Grupo de Investigación: Ecología y Genética de Poblaciones de Mamíferos, Instituto de Investigaciones Marinas y Costeras (IIMyC, CONICET), Universidad Nacional de Mar del Plata (UNMdP), Mar del Plata, Argentina

**Keywords:** *Ctenomys*, phylogeny, *talarum* group, Pampas region, conservation, *mendocinus* group, distributional boundaries, subterranean rodents

## Abstract

With about 68 recognized living species, subterranean rodents of the genus *Ctenomys* are found in a multiplicity of habitats, from the dunes of the Atlantic coast to the Andes Mountains, including environments ranging from humid steppes of Pampas to the dry deserts of Chaco region. However, this genus needs an exhaustive reevaluation of its systematic and phylogenetic relationships regarding the different groups that compose it. This knowledge is essential to propose biodiversity conservation strategies both at species level and at higher hierarchical levels. In order to clarify the taxonomy and the recent evolutionary history from populations of *Ctenomys* in the Pampas region, Argentina, phylogenetic relationships among them were evaluated using mitochondrial DNA sequences: gene encoding cytochrome *b* protein (1,140 bp) and the non-coding D-loop region (434 bp). To infer the divergence times inside the *Ctenomys* clade, a Bayesian calibrate tree using fossil remains data from different families within Caviomorpha was performed at first. Secondly, that calibration data was used as priors in a new Bayesian phylogenetic inference within the genus *Ctenomys*. This phylogenetic tree emphasized on species currently distributed on the Pampas region, more precisely considering both the *talarum* and *mendocinus* groups. Bayesian inferences (BI) were integrated with the results of a Maximum Likelihood approach (ML). Based on these results, the distributional limits of the *mendocinus* and *talarum* groups appear to be related to the physiognomy of the Pampas region soils. On the other hand, the validity of *C. pundti* complex as a differentiated species of *C. talarum* is debated. According to previous evidence from morphological and chromosomal studies, these results show a very low divergence between those species that originally were classified within the *talarum* group. Mitochondrial DNA sequences from populations associated with these putative species have not recovered as reciprocal monophyletic groups in the phylogenetic analyses. In conclusion, *C. talarum* and *C. pundti* complex might be considered as the same biological species, or lineages going through a recent or incipient differentiation process. The results obtained in this study have important implications for conservation policies and practices, since both species are currently categorized as Vulnerable and Endangered, respectively.

## Introduction

Subterranean rodents of the genus *Ctenomys* (Ctenomyidae: Caviomorpha; Blainville, 1826) are the most diverse in number of species of all native South American rodents, with about 68 recognized species (Bidau, 2015; Freitas, 2016; Teta and D’Elía, 2020; D’Elía et al., 2021). However, about 85 names have been assigned to biological entities of this genus and many taxa need to be properly delimited both geographically and systematically (Woods and Kilpatrick, 2005; Parada et al., 2011; Mapelli et al., 2017; Caraballo et al., 2020). In this context, the alpha taxonomy of *Ctenomys* has been intensively revised in the last decade, and new potential species are recurrently recognized (e.g., Parada et al., 2011; Caraballo and Rossi, 2017; Mapelli et al., 2017; Teta and D’Elía, 2020) and described (e.g., Freitas et al., 2012; Gardner et al., 2014; Teta et al., 2020; De Santi et al., 2021). Furthermore, many of the original classifications of species available in the literature have not been consistent with the geographic and taxonomic limits observed from new molecular information (e.g., Mapelli et al., 2017; Teta et al., 2020).

Regarding the origin of the genus, the fossil record suggests that it was approximately 3.5–3.8 million years ago (mya) (Reguero et al., 2007; Verzi et al., 2010; De Santi et al., 2021). Molecular estimates of divergence times to the most recent common ancestor (tMRCA) of *Ctenomys* species indicates an older origin for this group, most probably precluding 9.2 mya (range: 6.4–12.6 mya; see Parada et al., 2011). However, it should be noted a recent study using fossil calibrations within *Ctenomys* place the origin of this genus in a more recent period in relation to that reported by other authors (1.32 mya; range: 0.8–2.1; De Santi et al., 2021).

At the same time, the phylogenetic relationships between species and groups within the genus need an exhaustive reevaluation with novel morphological and molecular information; mainly for those groups considered of recent formation (see Mirol et al., 2010; Gómez Fernández et al., 2012; Caraballo et al., 2016; Mapelli et al., 2017). Parada et al. (2011) have identified eight major phylogenetic groups of species at the base of the *Ctenomys* tree (*boliviensis, frater, mendocinus, opimus, magellanicus, talarum, torquatus*, and *tucumanus*), most of them proposed previously based on external and internal morphology, karyology, and mitochondrial DNA analysis (see also Gardner et al., 2014; De Santi et al., 2021). In particular, the *talarum* group preferably occupies the Pampas region in Argentina and contains the species *Ctenomys talarum* Thomas, 1898 and *Ctenomys pundti* Nehring, 1900.

*Ctenomys talarum* is a species that, unlike many others of its genus, presents a broader distribution (although quite fragmented today) within the Pampas region, Argentina. This species occupies both sandy soils on the Atlantic coast of Buenos Aires province (Austral and Eastern Sand-dune Barriers; Isla et al., 1996; Isla and Lasta, 2010; Austrich et al., 2020a), and some minor inland areas (relictual and isolated populations) with increasingly harder, humid and vegetated soils far from the coast in Buenos Aires and La Pampa provinces (Malizia et al., 1991; Vassallo, 1998; Busch et al., 2000; Justo et al., 2003; Mora et al., 2006, 2007, 2013; Fernández et al., 2019). On the Atlantic coast, its distribution is roughly linear and their populations are more continuous than inland areas, where the presence of this species is highly patchy distributed. These relictual populations are characterized by small effective and census sizes (Mora et al., 2007, 2013; Fernández et al., 2019). Although declining in some portions of its distribution, *C. talarum* does not appear to be of range-wide conservation concern (Mora et al., 2013).

Also, in some coastal and inland areas, *C. talarum* coexists in close geographic proximity with some species belonging to the *mendocinus* group (*Ctenomys australis* Rusconi, 1934, *Ctenomys azarae* Thomas, 1903 and *Ctenomys porteousi* Thomas, 1919; see Contreras and Reig, 1965; Carnovale et al., 2017a, b; Cutrera and Mora, 2017; Mapelli et al., 2017; Churin et al., 2019). *Ctenomys talarum* is geographically sympatric with *C. australis* in the southern coastal region of Buenos Aires province (Contreras and Reig, 1965; Malizia et al., 1991; Vassallo et al., 1994; Vassallo, 1998; Busch et al., 2000; Justo et al., 2003; Mora et al., 2007; Cutrera and Mora, 2017; Austrich et al., 2020b), and parapatric with both *C. azarae* in the east of La Pampa province (García Esponda et al., 2009) and *C. porteousi* in the southwest of Buenos Aires province near Laguna Epecuén (Mora et al., 2013).

On the other hand, *Ctenomys pundti* occurs in the south of Córdoba and northeast of La Pampa provinces (Massarini, 1992; Tiranti et al., 2005). Currently, the distribution of this species complex is extremely reduced and highly fragmented, and is very close to those continental populations of *C. talarum* reported from La Pampa and north of Buenos Aires provinces (Fernández and Carnovale, 2019; Fernández et al., 2019). From reports of the possible extinction of this species in the type locality (Alejo Ledesma, Córdoba province) and, therefore, the extinction of the population that gives it its name, a nomenclature problem arose for the populations studied later, resorting to its assignment to what was called the *C. pundti* complex (Massarini, 1992; Tiranti et al., 2005).

Some studies have tried to clarify the evolutionary and systematic relationships between *C. talarum* and *C. pundti* complex analyzing some aspects of morphological variation, karyotype (Tate, 1935; Kiblisky and Reig, 1966), sperm morphology (Tiranti et al., 2005) and molecular phylogeny (Parada et al., 2011). These authors support the close phylogenetic relationship between *C. talarum* and the *C. pundti* complex, which definitely belong to the same evolutionary lineage.

Similar to many other species of this genus, there are still important ambiguities related to the geographic boundaries and taxonomic limits between *C. talarum* and *C. pundti* complex in the Pampas region of Argentina; for example, there is a lot of uncertainty whether both entities form suture zones or maintain areas of geographic sympatry.

Additionally, there is a lack of integrative molecular studies that have helped to clarify the phylogenetic relationships between *C. talarum* and *C. pundti* complex and their association with the other phylogenetic groups of *Ctenomys*; particularly with the Argentine species of its sister clade, the *mendocinus* group (i.e., *C. australis*, *C. azarae*, and *C. porteousi*), also named *mendocinus* species complex due particular ambiguities regarding its distributional and taxonomic boundaries (see Mapelli et al., 2017).

Although, *C. talarum* and the *C. pundti* complex (sensu Massarini, 1992) have traditionally been considered as distinct entities, their phylogenetic proximity, the similarity in morphological aspects of their phenotype, and geographical proximity between their populations suggest the possibility that both entities could be the same species.

Both species seem to be strongly affected by the environmental changes occurred in this area during the Pleistocene-Holocene boundary (Mapelli et al., 2017). The phylogeographic pattern recovered for *C. talarum* throughout its distribution range tells contrasting histories for different groups of populations (Mora et al., 2013). In addition, several inland populations in *C. talarum* seem to have become extinct recently (Quintana, 2004), while others persist in a highly fragmented landscape, associated to stream sand banks and inland areas with paleo-dunes (Mora et al., 2007, 2013; Cutrera and Mora, 2017; Fernández et al., 2019).

In this study, mtDNA sequences (D-loop and cytochrome *b*) were primary used in order to assess the phylogenetic relationships between the species included in the *talarum* group (*C. talarum* plus *C. pundti* complex). Secondly, the *talarum* group of species was compared with those associated with the *mendocinus* species complex on a broader phylogenetic context. This approach considers a sampling design in an extensive geographic area in the Pampas region, Argentina, and allows to establish the distributional boundaries of the species under study with greater accuracy.

## Materials and Methods

### Sampling Design

**FIGURE 1:**
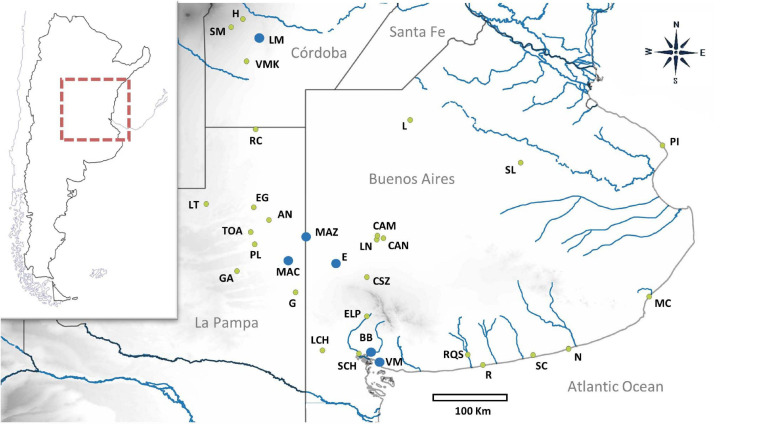
Geographical distribution of sampling locations is indicated with green dots for those previously studied and blue dots for those that are new. Abbreviations of each locality are as follows: H, Holmberg; SM, Sampacho; LM, Estancia Las Marianas; VMK, Vicuña Mackenna; RC, Realicó; L, Lincoln; SL, Saladillo; EG, El Guanaco; AN, Anguil; MAZ, Villa Maza; PL, Reserva Provincial Parque Luro; MAC, Macachín; E, Laguna Epecuén; G, Guatraché; CSZ, Coronel Suarez; ELP, Estancia La Paloma; PI, Punta Indio; MC, Mar de Cobo, RQS, Río Quequén Salado; SCH, Arroyo Sauce Chico; R, Balneario Reta; SC, San Cayetano; N, Necochea; VM, Villa del Mar; BB, Bahía Blanca, LN, La Navidad; CAM, Estancia La Manuela; CAN, Laguna Alsina; LCH, Laguna de Chasicó; LT, Luan Toro; GA, General Acha; TOA, Toay. The corresponding geographic coordinates are shown in Table 1.

Individuals of different putative species of *Ctenomys* were obtained from several field campaigns carried out in the following sampling sites: Realicó, Reserva Provincial Parque Luro, El Guanaco, Anguil, Guatraché, and Macachín in La Pampa province; Villa Maza, Lincoln, Saladillo, and Laguna Epecuén in Buenos Aires province; Estancia Las Marianas, Holmberg, Sampacho, and Vicuña Mackenna in Córdoba province (Figure 1). Field studies were performed between March 2014 and December 2017. Tissue samples (a very small portion of the tip of the tail for subsequent DNA extraction and genetic analyses) were obtained from captured individuals. These individuals were live trapped using Oneida Victor N°0 snap traps (Oneida Victor, Inc., Ltd., Eastlake, OH, United States), with a rubber cover to avoid injuring animals (Mora et al., 2006, 2010, 2016). Capture position were determined using a GPS. After collection of tissue samples for genetic analyses, animals were immediately released within the same burrow system where they had been captured. The handling of the individuals was carried out taking into account the guidelines of the “American Society of Mammalogists” (Sikes, 2016). Skin samples for each sampling location (Figure 1 and Table 1) were deposited in the Centro de Bioinvestigaciones (CeBio, UNNOBA-CICBA, Pergamino, Buenos Aires, Argentina) and preserved in 96% ethanol at −20°C until the time of their processing. Additionally, tissue samples from *C. talarum*, *C. azarae*, *C. porteousi*, *C. australis*, and *C. “chasiquensis”* were used. Those samples were obtained from different previous published studies (Mora et al., 2006, 2007, 2013, 2016; Mapelli et al., 2012, 2017) and are deposited in the Institute of Marine and Coastal Research (IIMyC, CONICET), Departamento de Biología, Facultad de Ciencias Exactas y Naturales, Universidad Nacional de Mar del Plata.

**TABLE 1:** List of localities from which DNA sequences were obtained in this study.

Sampling location	Code	Province	Species	Geographic coordinates	cyt-*b* (S)	D-loop (S)
Laguna Epecuén	E	Buenos Aires	*Ctenomys* sp.	37°12′41,51″S; 62°4959,69″W	4	4
Saladillo	SL	Buenos Aires	*Ctenomys talarum* (Massarini et al., 1995)	35°34′44,89″S; 59°39′57,07″W	1	1
Lincoln	L	Buenos Aires	*Ctenomys* sp. (Mora et al., 2013)	34°52′49,25″S; 61°34’31,93″W	7	7
Coronel Suarez	CSZ	Buenos Aires	*Ctenomys talarum* (Mora et al., 2013)	37°27′5,37″S; 61°52′22,89″W	7	3
Estancia La Paloma	ELP	Buenos Aires	*Ctenomys talarum* (Mora et al., 2013)	38°5′33,4″S; 62°19′33,2″W	3	3
Punta Indio	PI	Buenos Aires	*Ctenomys talarum* (Mora et al., 2013)	35°17′45,4″S; 57°12′51,6″W	4	–
Mar de Cobo	MC	Buenos Aires	*Ctenomys talarum* (Mora et al., 2013)	37°46′13,54″S; 57°26′41,53″W	4	–
Río Quequén Salado	RQS	Buenos Aires	*Ctenomys talarum* (Mora et al., 2013)	38°53′1,84″S; 60°19′14,53″W	2	1
Arroyo Sauce Chico	SCH	Buenos Aires	*Ctenomys talarum* (Mora et al., 2013)	38°42′10,5″S; 62°27′39,5″W	1	1
Balneario Reta	RQS	Buenos Aires	*Ctenomys talarum* (Mora et al., 2013)	38°53′5,79″S; 60°19′30,53″W	5	5
San Cayetano	SCH	Buenos Aires	*Ctenomys talarum* (Mora et al., 2013)	38°43′34,38″S; 59°26′33,29″W	2	1
Necochea	N	Buenos Aires	*Ctenomys talarum* (Mora et al., 2013)	38°37′21,8″S; 58°50′19,5″W	3	3
Villa del Mar	VM	Buenos Aires	*Ctenomys* sp.	38°50′55,7″S; 62°6′43,4″W	2	1
Bahía Blanca	BB	Buenos Aires	*Ctenomys* sp.	38°41′36,8″S; 62°15′23,7″W	3	1
Necochea	N	Buenos Aires	*Ctenomys australis* (Contreras and Reig, 1965)	38°37′26,9″S; 58°50′15,7″W	3	–
La Navidad	LN	Buenos Aires	*Ctenomys porteousi* (Mapelli et al., 2017)	36°50′10″S; 62°9′13,6″W	2	–
Estancia La Manuela	CAM	Buenos Aires	*Ctenomys porteousi* (Mapelli et al., 2017)	36°46′24,7″S; 62°8′28,6″W	1	–
Laguna Alsina	CAN	Buenos Aires	*Ctenomys porteousi* (Mapelli et al., 2017)	36°48′52,6″S; 62°2′16,9″W	1	–
Laguna de Chasicó	LCH	Buenos Aires	*Ctenomys “chasiquensis″* (Mapelli et al., 2017)	38°39′1,3″S; 63°5′30,8″W	3	–
Sampacho	SM	Córdoba	*Ctenomys pundti* complex (Tiranti et al., 2005)	33°21′40,60″S; 64°40′9,94″W	6	6
Holmberg	H	Córdoba	*Ctenomys pundti* complex (Tiranti et al., 2005)	33°13′46,29″S; 64°27’59,08″W	5	5
Estancia Las Marianas	LM	Córdoba	*Ctenomys* sp.	33°32′3,75″S; 64°10′9,34″W	6	6
Vicuña Mackenna	VMK	Córdoba	*Ctenomys pundti* complex (Tiranti et al., 2005)	33°21′41,89″S; 64°40′10,71″W	5	5
Realicó	RC	La Pampa	*Ctenomys pundti* (Tiranti et al., 2005)	35° 1′57,63″S; 64°14′46,23″W	4	4
El Guanaco	EG	La Pampa	*Ctenomys talarum* (Braggio et al., 1999)	36°18′34,24″S; 64°16′45,19″W	6	6
Guatraché	G	La Pampa	*Ctenomys pundti* complex (Tiranti et al., 2005)	37°41′59,48″S; 63°33′23,00″W	4	4
Macachín	MAC	La Pampa	*Ctenomys* sp.	37°12′22,32″S; 63°39′56,98″W	3	–
Anguil	AN	La Pampa	*Ctenomys azarae* (Tiranti et al., 2005)	36°30′7,99″S; 63°56′38,37″W	2	–
Reserva Provincial Parque Luro	PL	La Pampa	*Ctenomys azarae* (Massarini, 1992)	36°54′50,07″S; 64°15′45,13″W	7	–
Villa Maza	MAZ	La Pampa	*Ctenomys* sp.	36°47′9,05″S; 63°21′20,71″W	1	–
Luan Toro	LT	La Pampa	*Ctenomys azarae* (Mapelli et al., 2017)	36°15′13″S; 65°5′57,99″W	2	–
General Acha	GA	La Pampa	*Ctenomys azarae* (Mapelli et al., 2017)	37°21′7,4″S; 64°34′8,4″W	2	–
Toay	TOA	La Pampa	*Ctenomys azarae* (Mapelli et al., 2017)	36°42′50,2″S; 64°19′48,6″W	6	–

*Each of them with its abbreviated name (code), province to which it belongs, species to which individuals of said localities were assigned in previous studies (and their reference appointment), geographic coordinates and number of sequences (S) obtained for each locus.*

### DNA Extraction, Amplification, and Sequencing

DNA extraction was performed using the CTAB protocol (Doyle and Doyle, 1987). For the amplification of the complete cytochrome *b* (cyt-*b*) *locus* (1,140 bp), two pairs of primers were used (forward and reverse in both cases): MVZ 05 (5′-CGA AGC TTG ATA TGA AAA ACC ATC GTT-3′)/TUCO 06 (5′-GTG AAA TGG AAT TTT GTC TGA-3′) and TUCO 07 (5′-ATT ACA GCA ATA GTA ATA AT-3′)/TUCO14 (5′-CCA ATG TAA TTT TTA TAC-3′) (Smith and Patton, 1999; Wlasiuk et al., 2003). For the amplification of the partial 5′ hypervariable fragment of the mitochondrial control region (D-loop) (450 bp), a single pair of primers was used: TucoPro (5′-TTC TAA TTA AAC TAT TTC TTG-3′) and TDKD (5′-CCT GAA GTA GGA ACC AGA TG-3′), forward and reverse respectively (Kocher et al., 1989; Tomasco and Lessa, 2007). This was carried out through the polymerase chain reaction (PCR) method, which was prepared in a final volume of 20 μl, containing 25–100 ng of DNA, 1.5 mM of magnesium chloride, 0.2 μM of each primer, 0.2 mM of dNTPs, 1X reaction buffer, 0.5U of Taq T-Plus DNA polymerase (INBIO) and ultrapure sterile water.

The amplification conditions were as follows: initial denaturing at 94°C for 5 min, followed by 34 cycles, each one having a denaturing phase at 94°C for 45 s, one of the first binding to the template DNA (annealing) at 47°C for 45 s for all fragments equally and an extension phase at 74°C for 45 s for cyt-*b* and 1 min for D-loop; followed by 5 min at 74°C for a final extension and 10 min at 4°C. Amplified products were purified using 10U Exonuclease I (Thermo Scientific) and 1U FastAP Thermosensitive Alkaline Phosphatase (Thermo Scientific) incubated at 37°C for 15 min and then at 85°C for another 15 min to stop the reaction.

PCR products were sequenced through the ABI 3730XL automatic sequencer (Applied Biosystems) by MACROGEN (Korea) by direct sequencing.

A total of 117 tissue samples were processed in this work, the sample size for location and for *locus* is shown in Table 1.

### Editing, Alignment, and Sequence Analysis

A total of 117 sequences were obtained for the cyt-*b locus* and 71 sequences for the D-loop *locus*. Sequence electropherograms were visualized and inspected with Chromas 2.1.1 (Technelysium Pty Ltd, South Brisbane, Australia) and the two partially overlapping cyt-*b* fragments were aligned with Clustal *W* algorithm in MEGA6 (Tamura et al., 2013). These alignments were checked and edited manually.

In order to assess whether sequences retain the phylogenetic signal, the two resulting alignments were subjected to a saturation tests using DAMBE software (Xia and Xie, 2001), carrying out the test by Xia et al. (2003) in three different partitions: cyt-*b* first and second codon position, cyt-*b* third codon position and D-loop. This test estimates a sequence saturation index (Iss) and compares it to a critical saturation index (Iss.c) generated by a randomization process with 95% confidence. In this context, this test analyzes whether the observed Iss is significantly less than the estimated Iss (Iss.c). Sequences will be suitable for a phylogenetic study if this condition is met. IssSym is Iss.c assuming a symmetric topology, while IssAsym is Iss.c assuming an asymmetric topology, both topologies are taken into account. To select the best nucleotide substitution model, Akaike information criterion corrected for small samples (AICc) was used in jModelTest software (Posada, 2008) at three different sequence partitions: cyt-*b* first and second codon position, cyt-*b* third codon position and D-loop. The best model was estimated for each *locus* separately, and for both *loci* concatenated dataset, as required by each analyzes carried out in this work.

### Phylogenetic Analysis

#### Calibrated Trees

In order to carry out a more robust phylogenetic analysis and to get an accurate mutation rate of the *loci* used in this work, as well as to obtain ages of nodes that serve as seeds in future analyzes, two calibrated phylogenies were made.

Two phylogenetic independent trees with multiple calibration points (Table 2) were performed using BEAST 2.5.2 software (Bouckaert et al., 2019). The first phylogenetic tree was based on sequences of cyt-*b locus*, while the second one was based on a concatenated matrix of cyt-*b* and D-loop mitochondrial fragments. Sequences of all sampling locations studied here and sequences belonging to species of genus *Ctenomys* obtained from the GenBank database were combined. For this phylogenetic purpose, sequences of species of the suborder Caviomorpha and the African rodent *Thryonomys* were also used as outgroups (Table 3).

**TABLE 2:** Calibration nodes used in this study (A–F), ages are expressed in million years ago (mya), normal distribution was used in every node and publication reference of each one is shown.

Point	Node	mya	References
A	Crown Caviomorpha	33.8 ± 1.8	Vucetich et al., 1999; Opazo, 2005
B	Crown Cavioidea	27.9 ± 2.4	Opazo, 2005
C	Crown Octodontoidea	20.6 ± 2.4	Opazo, 2005
D	Octodontidae/Ctenomyidae split	13.5 ± 3.5	Opazo, 2005
E	Crown Caviidae	13 ± 1.5	Madozzo-Jaén and Pérez, 2017
F	Crown Octodontidae	7.79 ± 1.5	Opazo, 2005

**TABLE 3:** List of species for which a total of 128 sequences were obtained from the GanBank database, and used in phylogenetic analyzes.

	GenBank accession numbers	
Species	cyt-*b*	D-loop
*Ctenomys boliviensis*	AF007039, AF007040	JQ341037
*Ctenomys nattereri*	HM777484	JQ389117
*Ctenomys goodfellowi*	AF007050, AF007051	–
*Ctenomys erikacuellarae*	KJ778556, KJ778557	–
*Ctenomys steinbachi*	AF007043, AF007044	JQ341036
*Ctenomys opimus*	AF370701, AF007042	–
*Ctenomys fulvus*	AF370686, AF370688	–
*Ctenomys scagliai*	HM777494	–
*Ctenomys saltarius*	HM777493	–
*Ctenomys australis*	AF370697	–
*Ctenomys mendocinus*	AF370695, AF370695	MF770053, MF770064
*Ctenomys porteousi*	AF370681, AF370682	–
*Ctenomys flamarioni*	AF119107	JQ341041
*Ctenomys rionegrensis*	AF119114, AF538377	JQ341033, JX275652
*Ctenomys minutus*	HM777481, HM777482	HM236988, JX275648
*Ctenomys talarum*	HM777497, HM777498	–
*Ctenomys pundti*	HM777490, HM777490	–
*Ctenomys ibicuiensis*	JQ389020, JQ389023	JQ389115, JQ389116
*Ctenomys lami*	HM777477	JQ322898
*Ctenomys torquatus*	AF119109, AF119111	AY755460, JX275651
*Ctenomys roigi*	AF119111, AF119111	JQ686014, JQ686015
*Ctenomys perrensi*	HM777487, HM777488	JQ686016, JX275626
*Ctenomys dorbignyi*	JQ389030, JQ389030	JQ686025, JQ686026
*Ctenomys pearsoni*	JQ389030, HM777486	AY755457, AY755458
*Ctenomys maulinus*	AF370702, AF370703	–
*Ctenomys tucumanus*	AF370692, AF370694	–
*Ctenomys latro*	AF370704, HM777478	–
*Ctenomys occultus*	HM777485	–
*Ctenomys argentinus*	AF370680	JX275655
*Ctenomys colburni*	HM777474	–
*Ctenomys magellanicus*	DQ333326, HM777479	HQ262422, HQ262423
*Ctenomys haigi*	HM777476, KU659602	JQ341039
*Ctenomys coyhaiquensis*	AF119112, AF119113	–
*Ctenomys sericeus*	HM777496	–
*Ctenomys leucodon*	AF007056, HM544131	HM544131, NC020659
*Ctenomys tuconax*	AF370684, AF370693	–
*Ctenomys conoveri*	AF007054, AF007055	JX275654
*Ctenomys lewisi*	AF007049	–
*Ctenomys frater*	AF007045, KJ778558	–
*Ctenomys sociabilis*	HM777495, KU659601	HM544129, JQ341040
*Octodon degus*	AM407929	HM544134
*Spalacopus cyanus*	HM544133	NC020660
*Tympanoctomys barrerae*	AF007060	HM544132
*Cavia aperea*	GU136759	KT439327
*Capromys pilorides*	AF422915	FR686471
*Dactylomys dactylinus*	L23335	KU762015
*Dasyprocta leporina*	AF437808	AF437845
*Echimys didelphoides*	EU302705	EU313280
*Erethizon dorsatum*	FJ357428	–
*Galea musteloides*	GU082485	–
*Hydrochoerus hydrochoeris*	GU136721	EU149774
*Myoprocta acouchy*	AF437781	AF437816
*Octodontomys gliroides*	AF370706	KF917612
*Thryonomys swinderianus*	NC002658	NC002658

*GenBank accession numbers are also provided for both mtDNA regions (cyt-b and D-loop).*

Normal distribution was used for all calibration points, a relaxed lognormal clock model and calibrated birth-death branching rate (Heled and Drummond, 2014). For each data set (cyt-*b* and concatenated) two independent runs of 5 × 10^7^ MCMC generations (Markov chain Monte Carlo) were carried out, sampling every 5,000 generations. Data was partitioned into 1st + 2nd and 3rd codon position separately for cyt-*b* set. For concatenated data set, partitions were the same to the previous situation for cyt-*b*, with an additional partition for the D-loop mitochondrial fragment. Substitution model for each partition were estimated with jModelTest software (Posada, 2008) and used as priors. Software Tracer 1.7.1 (Rambaut et al., 2018) was used to determine the convergence of the posterior distribution, which was reached in all the runs (marginal distributions between runs were totally overlapped for all the parameters; the effective sample sizes “ESS” values were greater than 200 for both data sets). Log and trees files were combined using LogCombiner 2.5.2, trees were summarized with maximum clade credibility (MCC) from TreeAnnotator 2.5.2 software (Bouckaert et al., 2019) and a final tree was displayed in FigTree 1.4.4 (Rambaut, 2018).

Mutation rates and divergence times to the most recent common ancestors (tMRCA) of each taxonomic group were estimated and used later in the phylogenetic analyzes with species of the genus *Ctenomys.*

#### Phylogenetic Relationships Within *Ctenomys*

Subsequently, only for species of the genus *Ctenomys*, two Bayesian phylogenetic inferences (BI) were performed using BEAST 2.5.2 software (Bouckaert et al., 2019), one for concatenated data set and the other for cyt-*b* data set. For these phylogenetic inferences within *Ctenomys* cyt-*b* and D-loop mutation rates and tMRCAs calibrated in the previous analysis were used.

For cyt-*b* data set, two independent runs of 3 × 10^7^ MCMC generations were made, sampling every 3,000 generations and using two partitions: 1st + 2nd and 3rd codon position separately. For concatenated data set, two independent runs of 5 × 10^7^ MCMC generations were also made, sampling every 5,000 generations, using three partitions: 1st + 2nd and 3rd codon positions for cyt-*b*, and D-loop. The best model of nucleotide substitution for each partition was estimated with jModelTest (Posada, 2008). Mutation rates for these phylogenetic inferences were those previously estimated for each molecular marker in the previous analysis. Phylogenetic groups described in Parada et al. (2011) were defined as priors using the tMRCA obtained for these groups in the calibrated phylogenies of this study under a coalescing tree model. To determine convergence of the posterior distribution software Tracer 1.7.1 (Rambaut et al., 2018) was used. Log and trees files were combined using LogCombiner 2.5.2. After that, trees were summarized in Tree Annotator 2.5.2 software (Bouckaert et al., 2019) using the above-mentioned parameters and a final tree was displayed in FigTree 1.4.4 (Rambaut, 2018).

Also, two Maximum Likelihood (ML) phylogenetic inferences were performed, one to each dataset (cyt-*b* and concatenated), using the program MEGA6 (Tamura et al., 2013). Consistency of internal branches and nodes was evaluated with the standard bootstrap method (sampling with replacement, using 1,000 bootstrap replicates).

Divergence among pairs of species within *talarum* and *mendocinus* species groups of *Ctenomys*, and divergence among pairs of populations belonging to *talarum* group were estimated in MEGA6 (Tamura et al., 2013) using uncorrected p distance.

## Results

No stop codons, deletions or insertions were found in the alignment of cyt-*b* sequences used in the calibrated phylogenies, both for representatives of *Ctenomys* and for the rest of the hystricomorph rodents.

Xia’s test revealed that there is no substitution saturation in any of the three different partitions taken into account in both data sets: 1st + 2nd cyt-*b* codon positions, 3rd cyt-*b* codon position, and D-loop (see Supplementary Material 1).

### Calibrated Phylogenies

The phylogenetic relationships between the different families and superfamilies of the Order Caviomorpha were recovered with high posterior probability values (greater than 0.5, see Supplementary Material 2) for both data sets (cyt-*b* and concatenated). Topologies of both phylogenetic reconstructions were highly congruent. *Ctenomys* is a well-supported monophyletic clade, with a maximum posterior probability in both phylogenies.

Estimates of mutation rates from calibrated phylogenies were 0.0202 (SD: 0.0004) and 0.0295 (SD: 0.00029) substitutions per site per million years for cyt-*b* and D-loop, respectively. Divergence time to the most recent common ancestor (tMRCA) estimated for each group is shown in Table 4. It can be seen that 95% confidence intervals for each node overlap in the estimates of both data sets.

**TABLE 4:** Estimates of divergence times to the most recent common ancestor (tMRCA) for different taxonomic groups (nodes) inferred in each calibrated phylogeny.

Nodes	cyt-*b*		Concatenated (cyt-*b* + D-loop)	
	tMRCA (mya)	CI (95%)	tMRCA (mya)	CI (95%)
Caviomorpha origin	32.4	[29.2–35.6]	34.4	[31.1–37.6]
Cavioidea origin	26.9	[24.2–29.6]	26.9	[24.2–29.4]
Octodontoidea origin	20.5	[18–22.9]	19.6	[17.1–22.2]
Ctenomyidae/Octodontidae split	15.2	[12.5–17.8]	15.6	[12.5–18.4]
Caviidae origin	13.6	[12–15.4]	13.1	[11.4–14.8]
Octodontidae origin	8.3	[6.8–9.7]	8.0	[6.4–9.5]
Ctenomyidae origin	6.6	[4.7–8.7]	4.4	[2.9–6.3]

*Credibility intervals are also given in brackets.*

### Phylogenies of *Ctenomys*

**FIGURE 2:**
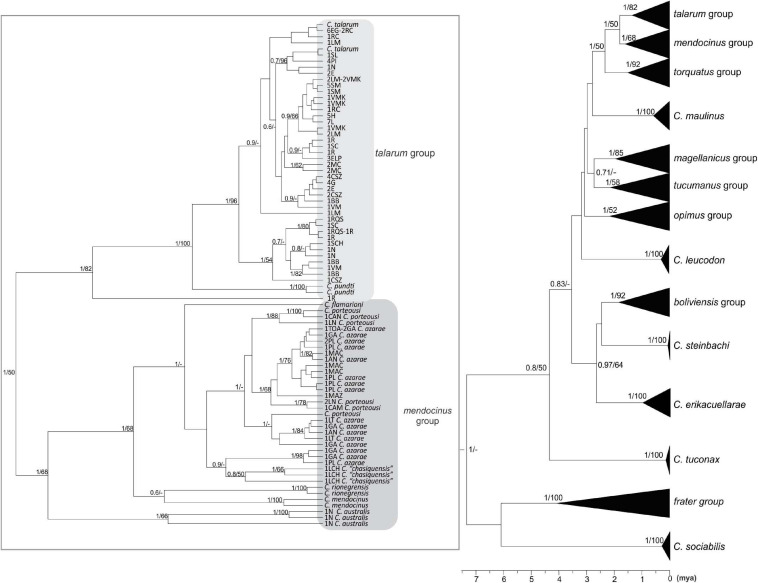
Phylogenetic tree using a Bayesian Inference from the complete cyt-*b* data set (1,140 bp). The numbers in nodes represent the values of node support in the Bayesian (between 0 and 1) and Maximum Likelihood (between 0 and 100) inferences. Values greater than 0.5 and 50 for Bayesian and Maximum likelihood inferences are shown, respectively. The bottom bar indicates the time from the root of the tree to the present time expressed in million years ago (mya). Clades belonging to *talarum* and *mendocinus* groups are shown in the left. Terminals represent different haplotypes, each with the abbreviations of populations to which it belongs, and its frequency, as well as the species to which it was previously assigned (if applicable). The abbreviations of sampling locations to which each sequence belongs are detailed in Table 1 and the detail of sequences extracted from GenBank database is indicated in Table 3.

**FIGURE 3:**
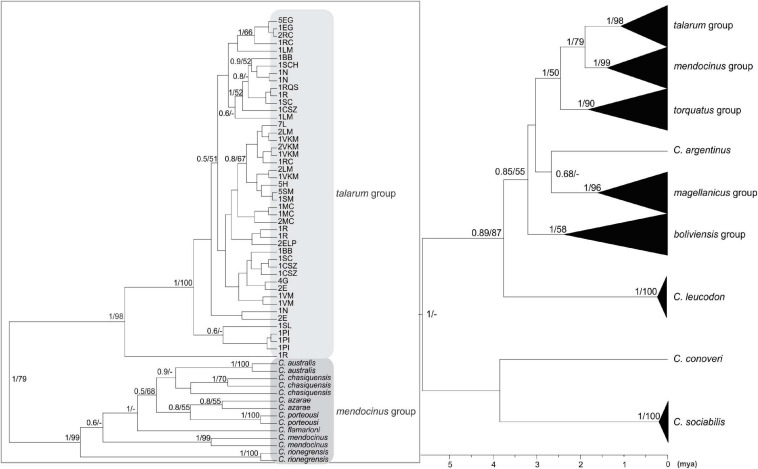
Bayesian inference phylogenetic tree based on concatenated dataset (1,574 bp). The numbers on the nodes represent the values of node support in the Bayesian (between 0 and 1; values higher than 0.5 are shown) and Maximum Likelihood (between 0 and 100; only values higher than 50 are shown) inferences. Bar at the bottom denotes the time from the root of the tree to the present time expressed in million years ago (mya). In the left part, clades belonging to *talarum* and *mendocinus* groups are shown. The terminals represent different haplotypes, each with the abbreviations of the sampling locations to which it belongs, and its frequency, as well as the species to which it was previously assigned (if applicable). The abbreviations of sampling locations are detailed in Table 1 and the detail of sequences extracted from GenBank database is indicated in Table 3.

The topologies obtained for both data sets, using mutation rates obtained for both *loci* from calibrated phylogenies, were highly concordant (see Figures 2, 3), with the exception of some phylogenetic groups that are not represented in the concatenated tree, due to the absence of sequences available in GenBank for D-loop in certain species (Table 3). These topologies were also very similar in both BI and ML reconstructions. The values of node support present in both inferences can be seen in Figures 2, 3.

The eight major phylogenetic groups reported by Parada et al. (2011) were supported by maximum posterior probability values using cyt-*b*. In concatenated phylogeny, D-loop sequences were only available to represent five of these eight groups (groups *talarum, mendocinus, torquatus, magellanicus*, and *boliviensis*), but were supported by high values of posterior probability either.

Some estimates of divergence times for these eight groups (Table 5) were more recent in both phylogenies than those obtained by Parada et al. (2011) and with much narrower confidence intervals.

**TABLE 5:** Divergence time to most recent common ancestor (tMRCA) inferred for different phylogenetic groups within *Ctenomys.*

	cyt-*b*		Concatenated (cyt-*b* + D-loop)	
Group	Mean (mya)	CI (95%)	Mean (mya)	CI (95%)
*talarum*	1.34	[0.89 – 1.82]	1.02	[0.59 – 1.48]
*mendocinus*	1.66	[1.17 – 2.17]	1.33	[0.90 – 1.82]
*boliviensis*	1.81	[1.13 – 2.51]	2.55	[1.48 – 3.69]
*frater*	3.02	[2.01 – 4.21]	-	-
*magellanicus*	2.01	[1.33 – 2.72]	1.65	[0.78 – 2.43]
*opimus*	2.17	[1.37 – 3.02]	-	-
*torquatus*	1.56	[1.06 – 2.06]	1.68	[1.16 – 2.27]
*tucumanus*	2.16	[1.45 – 2.89]	–	–

Samples collected in Realicó, El Guanaco, Guatraché, Lincoln, Saladillo, Laguna Epecuén, Estancia Las Marianas, Holmberg, Sampacho, and Vicuña Mackenna were grouped within the *talarum* group together with those analyzed samples of populations belonging to species *C. talarum* and *C. pundti* complex (Figures 2, 3). All these individuals showed similar external phenotypic morphological characteristics such as coat color, body weight, feet size and skull proportions (data not shown). On the other hand, samples from Villa Maza, Anguil, Reserva Provincial Parque Luro and Macachín were grouped within the *mendocinus* group, more closely related to the species *C. azarae* and *C. porteousi* (Figure 2).

Within the *talarum* group, *C. talarum* and *C. pundti* complex did not present reciprocal monophyly, more still, some ambiguities in the classification of internal clades associated previously to a particular species were observed.

The *mendocinus* group presents an ambiguous situation in this regard, some species such as *C. australis, C. mendocinus, C. rionegrensis* and the nominal species *C. “chasiquensis”* were monophyletic in both phylogenies, while *C. azarae* and *C. porteousi* only in the phylogeny performed using concatenated *loci*.

### Levels of Variation

At the interspecific level, genetic divergences between species belonging to *talarum* and *mendocinus* groups showed a range from 0.007 (divergence between *C. talarum* and *C. pundti* complex), to 0.052 (for the divergence between *C. flamarioni* and *C. pundti* complex (see Supplementary Material 3). These results denote that the comparisons of genetic divergence between *C. talarum* and *C. pundti* complex are one order of magnitude lower than the other interspecific comparisons.

The estimates of the genetic divergence obtained between pairs of populations belonging to the *talarum* group (*C. talarum* and *C. pundti* complex) ranged between 0.002 and 0.018 (see Supplementary Material 4). While the mean divergence obtained for populations associated with the *C. pundti* complex was 0.004, for *C. talarum* populations was 0.01 (Supplementary Material 4), one order of magnitude higher.

## Discussion

In order to provide further clarification on the distributional and taxonomic limits of the Pampas tuco-tucos from central Argentina, the phylogenetic relationships of individuals from populations traditionally associated with the *talarum* and *mendocinus* groups were studied. This study makes a substantial contribution in order to fill the distributional and taxonomic gaps of species distributed in the Buenos Aires, La Pampa, and southern of Córdoba provinces; which, in many cases, present disjunct and highly fragmented distributions in their distributional ranges. This study includes a large number of sequences of the complete cytochrome *b* gene (cyt-*b*) and partial D-loop of mitochondrial DNA from individuals sampled in an extensive area; with special emphasis on include those sites associated in previous studies with populations belonging to the *C. pundti* complex.

With the aim to have accurate estimates of divergence times of the main phylogenetic groups under study, fossil calibrations were performed. Thus, Bayesian calibration trees at the Caviomorpha and at the supra-generic level over *Ctenomys* allowed to obtain more precise estimates of mutational rates of the Family Ctenomyidae.

### Calibrated Phylogenies

The results derived from the calibrations allowed to compare the phylogenetic relationships among different groups of *Ctenomys* with the phylogenetic topologies reported in other studies where a similar number of species were used (see Parada et al., 2011).

This study results showed narrower confidence intervals of divergence times inferred for the nodes (Table 4), confirming that the inclusion of several calibration points, located adjacent in deep nodes of the phylogenetic tree, allows a greater accuracy in the estimation of divergence times (Linder et al., 2005; Mello and Schrago, 2013; Zheng and Wiens, 2015; Caraballo and Rossi, 2018). Both calibrated phylogenies showed consistent topologies and divergence times (with confidence intervals that overlap in all estimates) comparable to those published in previous studies (Opazo, 2005; Parada et al., 2011; Caraballo and Rossi, 2018).

An origin of approximately 6.6 mya was estimated for the crown of *Ctenomys* with a confidence interval (CI) between 4.7 and 8.7 mya in the phylogeny performed only with cyt-*b locus* (Table 4); while with concatenated data set the origin of *Ctenomys* was estimated in 4.4 mya (CI: 2.9 – 6.3; Table 4). Although the mean values of these nodes are different for each set of data, their confidence intervals overlap, so this difference would not be significant. On the other hand, the concatenated data set contains a smaller number of sequences within *Ctenomys*, this, added to the fact that the number of polymorphic sites is greater, could be the cause that the Bayesian inference shows different ages.

Confidence intervals of both phylogenetic inferences (cyt-*b* and concatenated) overlap each other and their estimates show an earlier origin for the genus than the obtained by Parada et al. (2011) with cyt-*b* (9.22; CI: 6.44 – 12.6 mya). It should be noted, however, Parada et al. (2011) considered a similar number of species but used a single calibration point (Caviomorpha – Wyss et al., 1993), unlike the six points used in this study (Table 2 and Supplementary Material 2).

In this regard, De Santi et al. (2021) reported a more recent origin for the genus (1.32 mya; CI: 0.8–2.1). In this study, fossil data within the Caviomorpha family but outside the crown *Ctenomys* were included, while De Santi et al. (2021) included a greater amount of fossil data, focusing on living and extinct species within this genus. Therefore, the origin of the genus *Ctenomys* estimated by these authors seems to be more limited considering all those fossil remains.

### Phylogenetic Inferences in *Ctenomys*

*Ctenomys* is the most diverse genus within Caviomorpha, but this diversity adds complexity when defining full species, both in terms of alpha taxonomy and phylogenetic relationships. In this study, the phylogenetic relationships of species traditionally associated with the *talarum* and *mendocinus* sister phylogenetic groups, considering a sampling design over an extensive portion of central Argentina were assessed. In this regard, two phylogenetic reconstructions were obtained from two mitochondrial data sets. These phylogenetic inferences yielded topologies with high posterior probability on the nodes for the main phylogenetic groups within *Ctenomys* (Figures 2, 3). This is consistent with results previously reported by other authors in *Ctenomys*. Both phylogenies were recovered with similar topologies, with higher posterior probability values than the obtained for species groups previously published by Parada et al. (2011), and comparable ages for the nodes of each group, being *torquatus*, *talarum*, and *mendocinus* those complex of species of more recent formation (see Table 5). In this context, this is the first study that extensively includes a large number of species using the cyt-*b* and D-loop mitochondrial fragments, integrating a wide geographic area in the Pampas region of Argentina.

In both approaches (BI and ML), *mendocinus* and *talarum* were sister phylogenetic groups. Sequences corresponding to all sampling locations addressed in this study (for both mitochondrial regions) were grouped into one or the other clade, with reciprocal monophyly observed between both phylogenetic groups. Unlike the above, not all the relationships between species within each phylogenetic group showed reciprocal monophyly.

Even though some species classically associated with the *mendocinus* group (Massarini et al., 1991; Cook and Lessa, 1998; Parada et al., 2011) showed reciprocal monophyly in the concatenated phylogenetic tree (Figure 3), this situation was not observed for *C. porteousi* and *C. azarae* (and partially by *C. “chasiquensis”* classified as nomen nudum) in the cyt-*b* phylogeny (Figure 2). These results may be due to the fact of including greater number of informative sites in the form of concatenated data, providing a more precise interpretation of the taxonomic limits between species of the *mendocinus* group. It should be noted, however, that cyt-*b* has proved to be very robust in several phylogenetic reconstructions of many groups of rodents, in particular within Caviomorpha (Cook and Lessa, 1998; Lessa and Cook, 1998; D’Elía et al., 1999; Mascheretti et al., 2000; Slamovits et al., 2001; Parada et al., 2011). In addition, it is essential to analyze the possibility of saturation in the D-loop mitochondrial region, which is a presumed neutral molecular marker with high rates of variability at population level. In this phylogenetic study, any signals of saturation in the D-loop sequences were observed. Beyond this and to avoid any inconvenience related to saturation, the partial D-loop sequences concatenated with cyt-*b* were used.

On the other hand, the lack of reciprocal monophyly for several existing nominal forms may reflect a mismatch between the gene tree and the species tree due to the fixation of alternative ancestral haplotypes, which would lead to incomplete lineage sorting (Neigel and Avise, 1986). The lineage sorting is responsible for eliminating these ancestral polymorphisms over time, then sister species eventually become monophyletic. However, this process can occur incompletely when the speciation process is faster than the stochastic sorting of polymorphisms within each lineage (Sullivan et al., 2002). Under conditions of rapid speciation, large effective population sizes are needed to reduce genetic drift effects and decrease lineage sorting rates (Parker and Kornfield, 1997).

### Populations of *mendocinus* Group

Sampling locations of Reserva Provincial Parque Luro, Anguil, Macachín, and Villa Maza grouped within the *mendocinus* clade in the phylogeny carried out with the cyt-b *locus* (these were sequenced only for this molecular marker: see Table 1) with a maximum posterior probability and greater affinity with *C. azarae* and *C. porteousi*. These results are partially in disagreement with those reported by D’Elía et al. (2021), who proposed that *C. azarae* and *C. porteousi* should be considered as subjective junior synonyms of *C. mendocinus*.

*Ctenomys azarae* and *C. porteousi*, together with *C. australis*, *C. flamarioni* Travi, 1981, *C. mendocinus* Philippi, 1869, and *C. rionegrensis* Langguth and Abella, 1970 are included in the *C. mendocinus* species group (Massarini et al., 1991; D’Elía et al., 1999; Parada et al., 2011) or *C. mendocinus* species complex (Mapelli et al., 2017). Even though the distinction of *C. azarae* and *C. porteousi* from *C. mendocinus* has been largely questioned in the literature, some advances have been made on the subject in relation to phylogenetic and phylogeographic aspects (Parada et al., 2011; Mapelli et al., 2017). In fact, results of the phylogenetic reconstructions made by Mapelli et al. (2017) show the presence of three major clades into the *C. mendocinus* species complex, which do not correspond to the classically proposed taxonomy or the distributional limits previously assumed for many of those species. Beyond this, these authors recognize the need for more in-depth comprehensive studies regarding aspects of morphological and molecular taxonomy. On the other hand, D’Elía et al. (2021) did not mention some attributes of the natural phenotypic variation (e.g., body sizes; pelage colors) and the conformation of major phylogeographic groups highlighted in Mapelli et al. (2017).

Currently, new species are being described for the group *mendocinus*, expanding the geographical range in which it is possible to find this group (see Tammone and Pardiñas, 2021). Possibly, the *C. mendocinus* species group is a complex of recently formed species, which requires exhaustive morphological and genomic approaches to more accurately assess the distributional and taxonomic limits of the species that have been classically associated with this group. In this sense, the present approach only attempts to determine the assignments of individuals to particular phylogenetic groups. It should be noted, however, that monophyly in all species described as belonging to the *C. mendocinus* species complex were obtained by increasing the level of polymorphism with concatenated mitochondrial *loci* (cyt-*b* and D-loop).

In this context, habitat configuration of these sampling locations in Reserva Provincial Parque Luro, Anguil, Macachín, and Villa Maza (Figure 1) are very similar to that observed in other species belonging to the *mendocinus* group, which is characterized mainly by sandy and more arid lands, with low vegetation cover (Mapelli et al., 2017), compared to harder and vegetated soils preferred by individuals of coastal and continental species of the *talarum* group (Vassallo, 1998; Busch et al., 2000; Cutrera et al., 2006, 2010; Mora et al., 2013).

The central-western area of the Pampas region is characterized by the presence of sand-dunes, shaped like the fingers of a hand, and interspersed with low and more vegetated soils with a higher percentage of humus (Cano et al., 1980; Salazar Lea Plaza, 1980). It is precisely in these sectors of higher substrate of sand-dunes where these populations are located, which were subsequently grouped phylogenetically within the *mendocinus* group. Those populations located in the adjacent soils (characterized by lower soils, with greater hardness and vegetation cover, and in some cases with high levels of water logging) to these higher sand-dune areas, such as Guatraché, El Guanaco and Laguna Epecuén, were included within the *talarum* group.

These results show a high specificity of each group of species in terms of the occupation of different habitats. In particular, soil characteristics could be a good predictor of the occurrence of each species of *Ctenomys* from different phylogenetic groups (see Mora et al., 2016, 2017 for the nominal form *C. ”chasiquensis*”; see Mapelli et al., 2012, 2017 for *C. azarae* and *C. porteousi*). Such specificity in occupation of different environment configurations goes beyond geographical proximity, since, in many regions, species of different phylogenetic groups are very close to each other (e.g., Macachín and Guatraché; see Figure 1).

### Populations of *talarum* Group

Sampling locations Holmberg, Sampacho, Estancia Las Marianas, Vicuña Mackenna, Realicó, El Guanaco, Guatraché, Laguna Epecuén, Saladillo, and Lincoln grouped within the *talarum* group with a maximum posterior probability (Figures 2, 3).

Some of these populations were previously assigned, through karyotype and sperm morphology studies, to the *C. pundti* complex (see Massarini, 1992; Tiranti et al., 2005) and not directly to *C. pundti*. These authors preferred this designation due to *C. pundti* is extinct in its type locality (Alejo Ledesma, Córdoba province) and individuals from Puente Olmos (the closest studied location to the type locality of the species) presented the most differentiated karyotype of all those addressed in that study (see Tiranti et al., 2005). However, this species is currently extinct from Puente Olmos (personal observation). After a very exhaustive inspection of the study area, the presence of some form of this complex of species was registered in Estancia Las Marianas, the closest locality to the Alejo Ledesma (approximately 140 km).

Ambiguities between the species *C. talarum* and *C. pundti* complex observed in this study with molecular data were also reported in previous studies, including some of the populations discussed here. Massarini (1992) suggest that the populations of Realicó, El Guanaco, Guatraché, and Vicuña Mackenna were closely related from the chromosomal perspective and, at the same time, the degree of differentiation between those and *C. talarum* populations was very slightly both at the chromosomal and morphological level. These authors suggest that one karyotype, belonging to a population from Puente Olmos in Córdoba province (2n = 50), could be a derived form with respect to the other populations of *C. pundti* complex (2n = 44–50); since the karyotypes found in the other populations, except for a few rearrangements, were shared with *C. talarum* (2n = 46–50).

On the other hand, Justo (1992) describes the subspecies *C. talarum occidentalis* only based on morphological data. Among the samples used in this description there were individuals from El Guanaco, sampling location that was later reassigned to *C. talarum talarum* (Braggio et al., 1999) because its chromosomal similarity with samples described in Massarini et al. (1995) from Saladillo, Buenos Aires province. Subsequently, populations from El Guanaco were reassigned to *C. pundti* complex by Tiranti et al. (2005), together with the other populations addressed in the study of Justo (1992).

Populations of *Ctenomys* studied by Tiranti et al. (2005), belonging to the localities of Realicó, El Guanaco and Guatraché, turned out to be very close to each other from the karyotypic point of view; while Holmberg and Sampacho were indistinguishable from each other, and different from the previous three populations on the basis of a single Robertsonian change. In that study, based on evidence such as chromosomal homology and sperm morphology, the authors suggest that *C. talarum* and *C. pundti* complex are lineages in a process of recent diversification.

In this context, three subspecies are described for *C. talarum*: *C. t. talarum* Thomas, 1898 with type locality at Los Talas (Buenos Aires province); *C. t. recessus* Thomas, 1912 with the type locality referred to Bahía Blanca (Buenos Aires province); and *C. t. occidentalis* Justo (1992) with type locality at Luan Toro (La Pampa province) (see Justo et al., 2003). These authors suggest that the town of Santa Clara del Mar is the southernmost limit of *C. talarum talarum*, with a gap between this town and Necochea, where there is no presence of tuco-tucos at present. Contreras and Reig (1965) described the distribution of *C. t. recessus* between Necochea (Parque Miguel Lillo) and Bahía Blanca. Thus, *C. t. talarum* and *C. t. recessus* are distributed along the coast of Buenos Aires province, while *C. t. occidentalis* occurs only in central areas of La Pampa province. In this study it was possible to verify that the genetic distances between pairs of populations assigned to the regions of occurrence of those subspecies are low (0.01–0.015) and similar to the existing genetic distances between these populations and those previously assigned to *C. pundti* complex (0.003–0.012) (see Supplementary Material 4).

Additionally, the genetic divergence between *C. talarum* and *C. pundti* complex was low and within the order of the average distances between the *C. pundti* complex populations (see Supplementary Materials 3, 4). Divergences between *C. talarum* or *C. pundti* complex and species belonging to *mendocinus* group are at least one order of magnitude greater than the divergence *C. talarum–C. pundti* complex (Supplementary Material 3).

It should be noted, lineages that exhibit profound phylogenetic divergence and reach reproductive isolation generally show reciprocal monophyly in gene trees, allowing to observe clear boundaries at geographical and taxonomic levels. On the other hand, lineages that have recently diverged gathered very little divergence time, are often problematic in order to obtain robust systematic delimitations (Shaffer and Thomson, 2007). In addition, the retention of ancestral polymorphisms between different lineages increases the time required to achieve reciprocal monophyly in gene trees (Rosenberg, 2002). In these cases, the gene trees, at least using a single *locus*, are not sufficient to draw clear conclusions regarding the taxonomy of these groups.

That issue is evidenced in many complexes of emergent species within respective phylogenetic groups of *Ctenomys* (Caraballo et al., 2016; Caraballo and Rossi, 2017). In addition to the *talarum* group, the *mendocinus* species complex also presents great ambiguities regarding classification and assignment of individuals from different geographic regions to particular full species (Mapelli et al., 2017; Tammone and Pardiñas, 2021). In this context, there are some difficulties to correctly assign cryptic species. The accurate identification of populations is another problem in a context of divergence of a species complex. Given that tuco-tucos tend to have high specificity in the habitat use and landscape requirements (Busch et al., 2000), the lack of information about the levels of taxonomic diversification would lead to underestimate the impact of different threats. This has direct impacts on conservation decision and management.

In this sense it is necessary to observe that extensive areas of the central region of Argentina were originally characterized by natural grassland systems with a predominance of sandy environments in the Monte and Espinal ecoregions. At present, agricultural activity in the Pampas region has caused deep degradation and removal of the original natural habitats which has increased the fragmentation of grassland and wetlands and made of many of them disappear (Quirós et al., 2002; Brandolin et al., 2013). It should be noted that in a very few decades the landscape in the Pampas region was highly transformed in an accelerated way, greatly increasing the fields of cultivation, especially the soybean planting in sectors like the south of Córdoba province (Brandolin et al., 2013).

The Caldenal ecosystem in La Pampa province also has undergone substantial changes in land use and cover during the last century due to anthropic activities, which have increased the areas covered by agriculture and woody vegetation; changing an original landscape of savannas with little vegetation cover to a mosaic of dense forests interspersed with intensive agriculture (González-Roglich et al., 2015).

These changes have caused the loss of biodiversity for many native species and the acceleration of habitat fragmentation in the case of tuco-tucos populations (Tiranti et al., 2005; Mora et al., 2017; Mapelli et al., 2017). The species *C. talarum* and *C. pundti* were recently classified as Vulnerable (VU) and Endangered (EN) respectively, with dune afforestation, agriculture, floods and habitat fragmentation as the main triggers (Fernández et al., 2019; Fernández and Carnovale, 2019). Therefore, a schedule of conservation of tuco-tucos and mammals in general from central Argentina requires the combined action of protecting selected grasslands representative of the diversity of regional habitats and the urgent implementation of management actions at the regional scale.

Taking into account all this information, there is a possibility that the populations that are currently found in the south of Córdoba and north of La Pampa provinces could be remnants of a broader original distribution of the *C. talarum* species, or the product of a later colonization of said species in the study region.

Finally, and as a main conclusion, the results of this study are consistent with previous evidence showing that divergence of species within the *talarum* group is minimal. Considering the evidence provided by molecular data sets used in this study, it is suggested that *C. talarum* and *C. pundti* complex could be considered as the same biological species, or lineages going through a recent or incipient differentiation process. To confirm any of these hypotheses, a multidisciplinary approach will be necessary, such as a study of the type material of each species (Stolz et al., 2013; Tammone et al., 2016; Sánchez et al., 2019), deep chromosome studies (Castilho et al., 2012; Kubiak et al., 2020), wider multi-*locus*/genomic analyses (MacManes et al., 2015; Lopes et al., 2020), environmental and ecological features (Kubiak et al., 2017; Lopes et al., 2020), and mainly exhaustive approaches on phenotypic variation at different levels (Freitas et al., 2012; Gonçalves, 2021), in conjunction with complex and deeper morphometric analyzes that integrate classical and geometric morphometry (Borges et al., 2017; Fornel et al., 2018; Leipnitz et al., 2020).

## Data Availability Statement

The datasets presented in this study can be found in online repositories. The names of the repository/repositories and accession number(s) can be found below: GenBank accession numbers: MZ332974–MZ333091.

## Ethics Statement

The animal study was reviewed and approved by Comité Institucional de Cuidado y Uso de Animales de Laboratorio (CICUAL) – Facultad de Ciencias Exactas y Naturales de la Universidad Nacional de Mar del Plata.

## Author Contributions

CC processed the samples, analyzed the data, and wrote the manuscript. GF and MSM contributed the reagents and materials, helped with the data analysis, and provided guidance on the whole manuscript. All the authors participated in the sample collection and, as well as also reviewed and approved the final submission.

## Conflict of Interest

The authors declare that the research was conducted in the absence of any commercial or financial relationships that could be construed as a potential conflict of interest.

## Publisher’s Note

All claims expressed in this article are solely those of the authors and do not necessarily represent those of their affiliated organizations, or those of the publisher, the editors and the reviewers. Any product that may be evaluated in this article, or claim that may be made by its manufacturer, is not guaranteed or endorsed by the publisher.

## Supplementary Material

Supplementary Material 1 Saturation tests for each partition used in phylogenetic analysis.

Supplementary Material 2 Calibrated phylogenetic trees. **(A)** Phylogenetic reconstruction using cyt-*b* mitochondrial data (1,140 bp), **(B)** phylogenetic reconstruction performed from concatenated data (1,574 bp). Letters A to G indicate the calibration points previously detailed in Table 2, and numbers in each node indicate the posterior probability value. The bottom bar indicates the time from root of the tree to the present time, expressed in million years ago (mya).

Supplementary Material 3 Table of genetic distances between species of the *talarum* and *mendocinus* groups.

Supplementary Material 4 Table of genetic distances between populations within the *talarum* group.

Supplementary Material 5 Table of haplotypes of D-loop *locus* sequenced for this work, with their frequencies in the corresponding sampling locations, species group and GenBank accession numbers.

Supplementary Material 6 Table of haplotypes of cyt-*b locus* sequenced for this work, with their frequencies in the corresponding sampling locations, species or species group and GenBank accession numbers.
