# Supplementary material for: Redefining the Distributional Boundaries and Phylogenetic Relationships for Ctenomids From Central Argentina
Source: Front Genet. 2021 Aug 4;12:698134. doi: 10.3389/fgene.2021.698134 (PMC8372524; doi:10.3389/fgene.2021.698134)
Supplement: Supplementary Material 1 — Saturation tests for each partition used in phylogenetic analysis. [file Data_Sheet_1.docx]

**Supplementary material**

Saturation tests for each partition used in phylogenetic analysis.

**cyt-b data set**

1^st^ + 2^nd^ codon position

| Num OTU | Iss | IssSym | T | DF | P | IssAsym | T | DF | P |
| --- | --- | --- | --- | --- | --- | --- | --- | --- | --- |
| 4 | 0,046 | 0,810 | 99,554 | 759 | 0,000 | 0,779 | 95,493 | 759 | 0,000 |
| 8 | 0,050 | 0,773 | 87,832 | 759 | 0,000 | 0,665 | 74,655 | 759 | 0,000 |
| 16 | 0,056 | 0,755 | 82,164 | 759 | 0,000 | 0,548 | 57,867 | 759 | 0,000 |
| 32 | 0,060 | 0,728 | 78,547 | 759 | 0,000 | 0,408 | 40,854 | 759 | 0,000 |

3^rd^ codon position

| Num OTU | Iss | IssSym | T | DF | P | IssAsym | T | DF | P |
| --- | --- | --- | --- | --- | --- | --- | --- | --- | --- |
| 4 | 0,132 | 0,797 | 46,411 | 523 | 0,000 | 0,764 | 44,083 | 523 | 0,000 |
| 8 | 0,139 | 0,754 | 39,371 | 523 | 0,000 | 0,642 | 32,540 | 523 | 0,000 |
| 16 | 0,146 | 0,725 | 37,862 | 523 | 0,000 | 0,515 | 24,144 | 523 | 0,000 |
| 32 | 0,147 | 0,705 | 35,915 | 523 | 0,000 | 0,378 | 14,872 | 523 | 0,000 |

**Concatenated data set**

cyt-b 1^st^ + 2^nd^ codon position

| Num OTU | Iss | IssSym | T | DF | P | IssAsym | T | DF | P |
| --- | --- | --- | --- | --- | --- | --- | --- | --- | --- |
| 4 | 0,064 | 0,810 | 81,304 | 759 | 0,000 | 0,779 | 77,906 | 759 | 0,000 |
| 8 | 0,058 | 0,773 | 79,261 | 759 | 0,000 | 0,665 | 67,231 | 759 | 0,000 |
| 16 | 0,065 | 0,755 | 73,516 | 759 | 0,000 | 0,548 | 51,486 | 759 | 0,000 |
| 32 | 0,068 | 0,728 | 70,745 | 759 | 0,000 | 0,408 | 36,363 | 759 | 0,000 |

cyt-b 3^rd^ codon position

| Num OTU | Iss | IssSym | T | DF | P | IssAsym | T | DF | P |
| --- | --- | --- | --- | --- | --- | --- | --- | --- | --- |
| 4 | 0,326 | 0,787 | 20,989 | 379 | 0,000 | 0,756 | 19,599 | 379 | 0,000 |
| 8 | 0,355 | 0,740 | 17,631 | 379 | 0,000 | 0,629 | 12,554 | 379 | 0,000 |
| 16 | 0,366 | 0,698 | 16,301 | 379 | 0,000 | 0,515 | 6,024 | 379 | 0,000 |
| 32 | 0,370 | 0,689 | 15,957 | 379 | 0,000 | 0,414 | 1,955 | 379 | 0,0071 |

D-loop

| Num OTU | Iss | IssSym | T | DF | P | IssAsym | T | DF | P |
| --- | --- | --- | --- | --- | --- | --- | --- | --- | --- |
| 4 | 0,221 | 0,791 | 26,012 | 433 | 0,000 | 0,758 | 24,515 | 433 | 0,000 |
| 8 | 0,230 | 0,745 | 21,015 | 433 | 0,000 | 0,634 | 16,645 | 433 | 0,000 |
| 16 | 0,251 | 0,709 | 16,822 | 433 | 0,000 | 0,499 | 9,125 | 433 | 0,000 |
| 32 | 0,276 | 0,695 | 13,575 | 433 | 0,000 | 0,367 | 2,943 | 433 | 0,0034 |
