# Supplementary figures and images for: Redefining the Distributional Boundaries and Phylogenetic Relationships for Ctenomids From Central Argentina
Source: Front Genet. 2021 Aug 4;12:698134. doi: 10.3389/fgene.2021.698134 (PMC8372524; doi:10.3389/fgene.2021.698134)

A

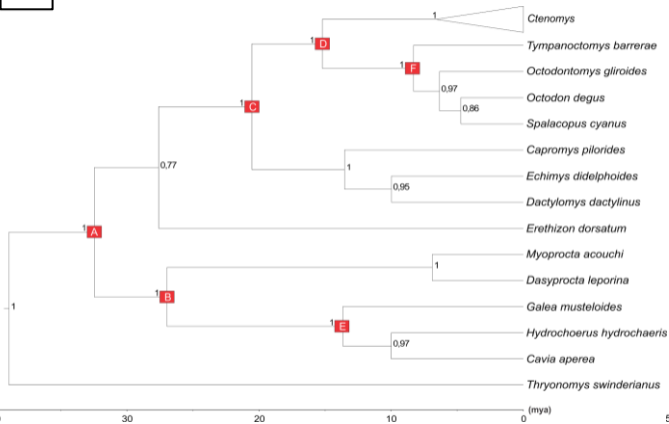

B

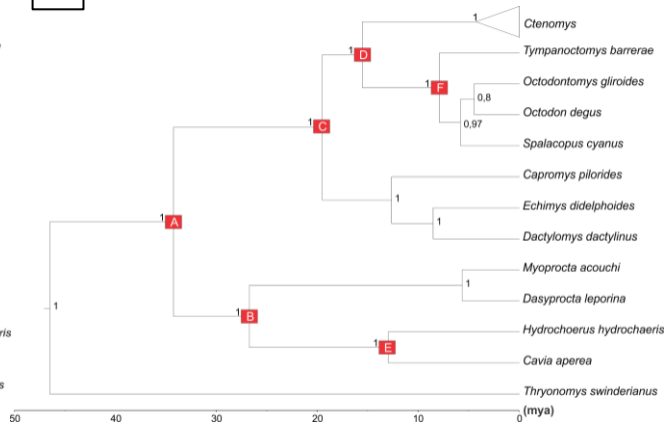

Supplement: Supplementary Material 2 — Calibrated phylogenetic trees. (A) Phylogenetic reconstruction using cyt-b mitochondrial data (1,140 bp), (B) phylogenetic reconstruction performed from concatenated data (1,574 bp). Letters A to G indicate the calibration points previously detailed in Table 2, and numbers in each node indicate the posterior probability value. The bottom bar indicates the time from root of the tree to the present time, expressed in million years ago (mya). [file Data_Sheet_2.pdf]

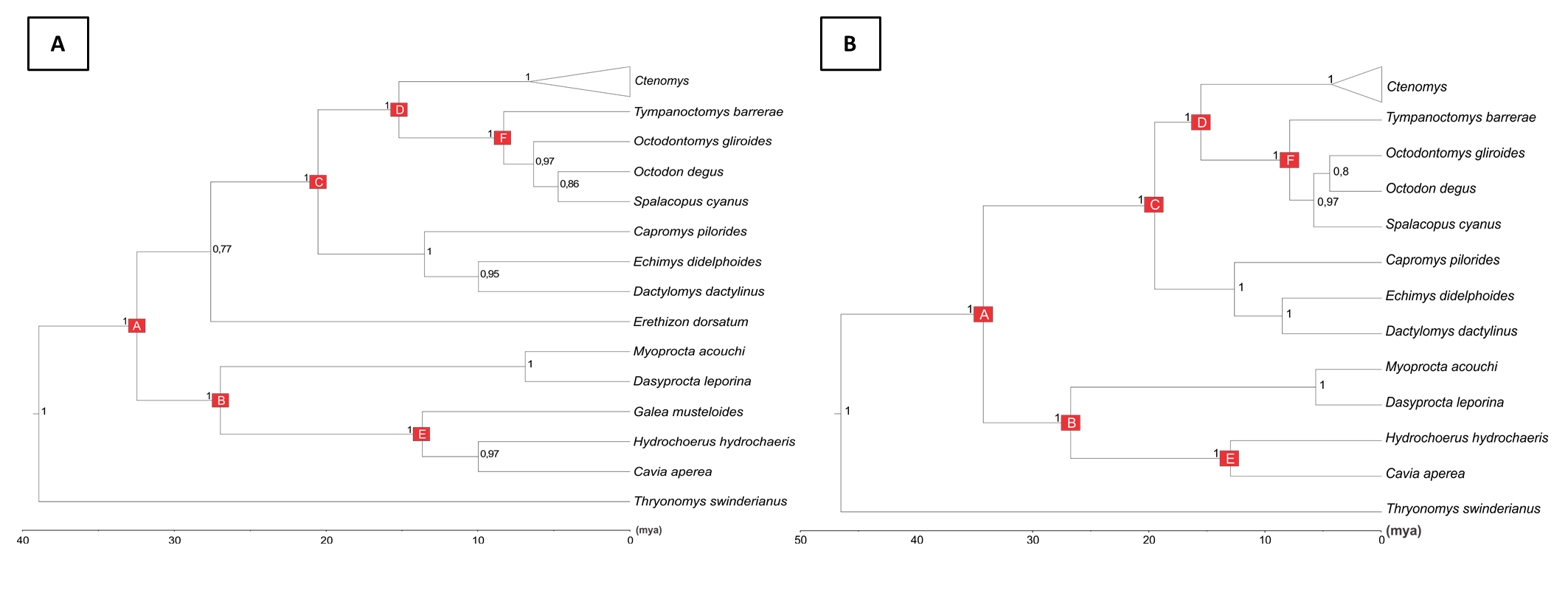

Supplement: Supplementary file 7 [file Image_1.TIF]
